# Hospital Meetings, &c.

**Published:** 1899-06-24

**Authors:** 


### HOSPITAL MEETINGS, &C.

### ROYAL EYE HOSPITAL.

### Opening of the Stock Exchange Ward.

Mr. J. K. Hichens, chairman of the Stock Exchange, accompanied by Mr. W. M. Chinnery and other members of that institution, in addition to many other ladies and gentlemen, opened on the 6th inst. one of the hitherto unoccupied wards of the Royal Eye Hospital, Southwark, and which, in recognition of the handsome support accorded to the hospital by members and their friends, is to be called the Stock Exchange Ward.

Professor McHardy, the senior surgeon, on behalf of the council, asked Mr. Hichens to declare the ward open, and briefly reviewed the history of the institution. It was inaugurated, he said, in 1857, but the premises were most unsuitable, and were demolished in 1889. The council then nominated him as a committee of one to superintend the rebuilding, thus favouring continuity of ideas, and the possibility of making the most of opportunities. The site was unequalled for its purpose. It was situated in the geographical centre of London; in the middle of one of the very poorest districts of the metropolis; and with six great thoroughfares converging on it with tramlines, making it easily accessible to all parts south of the Thames. The number of new patients had increased from 6,000 in 1890 to 17,118 last year, and experience had shown that they could count on increased support for a hospital which they could claim was up-to-date, and a model of convenience, completeness, sanitation, efficiency, and economy. They had aimed at, and he believed successfully, the completest possible severance of the out-patient department frcm the in-patient, with facilities of access between the two, but with a minimum risk of contamination; compactness, so as to reduce working expenses; abundance of cubic space, light, air, ventilation, and warmth; avoidance of workhouse-like arrangements so distasteful to the poor; and restriction of outlay on official and staff accommodation so far as was compatible with due regard to the brightness, wholesomeness, and well-being of those who had to minister to the patients. What they were there for that day would help still further to remove from South London the blot of the great disparity—one in five—between the hospital beds available as compared with London north of the Thames.

Mr. Hichens, in declaring the ward open, said Mr. McHardy's reference to the generosity of the members of the Stock Exchange to that hospital might be due to the fact that as members of that institution they had to be very wideawake. No form of affliction caused so much sympathy as anything which had to do with the eyes. Many of them had, at one time or another, taken part in a discussion whether it was better to be deaf or blind. Most people seemed to think that it was better to be deaf, but it was nevertheless a fact that people suffering from blindness were generally cheerful. This cheerfulness was almost entirely due to the great sympathy which everyone had for those who had afflictions of the eyes. The statement that an operation was to be performed on the Queen's eyes for the removal of cataract had, he saw, been questioned, but if it were true, they had now this consolation, that with the advance of science the operation would be an absolutely painless one, and with a practical certainty of success. They would all, he felt sure, be delighted to learn that Her Majesty had been restored to the enjoyment of her eyesight. (Applause.)

---

### ROYAL ORTHOPÆDIC HOSPITAL.

### Alterations in the Laws.—Resignation of the President and Five Members of the Committee.

On Thursday, 8th inst., a meeting of the Royal Orthopædic Hospital was held in the Royal Medical and Chirurgical Society's Rooms, Hanover Square, having been requisitioned by Sir Walter Gilbey, and the members of last year's committee elected in March to serve on this year's committee. Lord Wantage, the president of the institution, occupied the chair.

Sir Walter Gilbey proposed that in Law II., line 8, the word "twelve" should be substituted for the word "six," thus providing that no annual governor should vote at any election unless his first annual subscription had been paid twelve months previously to the day of election.

Sir Ernest Clarke seconded the resolution. He said the object of this amendment was to reaffirm and strengthen a law of the hospital which had now been in force in the hospital for over 41 years; a bye-law recognised by all hospitals as necessary and desirable, and which, if it had been obeyed by the new governors who mustered in such numbers at the last annual court, would have avoided the present crisis in the hospital's affairs. The law was obviously designed to protect the regular subscribers to the charity from what Mr. Alderman Bell had well called a raid, made by persons who subscribe (or have subscribed for them) one single subscription, in order to qualify them for a vote at the annual court of the hospital. It was admitted in a circular issued by the other side that the very great majority of those who overcrowded the small board-room on March 27th were elected governors since last December. They were therefore not qualified under the existing Law II., which limits the right of election to the committee to those who have been on the books six months. The earliest of them did not send in their postal orders till December, and he who confessed himself in his recent circular the arch organiser, Mr. Bernard Parker, did not himself declare himself as a would-be subscriber till towards the end of February. He (the speaker) was too ill to attend the annual court himself, but he raised the question as to the legality of the voting on that occasion at the earliest opportunity open to him, viz., at the first meeting of the new committee on April 21st. He then pointed out that six of the eight new committeemen were elected by the votes of those who were not qualified to vote at all, and that the two remaining members of the committee, and these the most powerful, were not even governors of the hospital at all, not being on the books when the election took place. No attempt was, or indeed could be, made to justify this last infraction of the laws—the oppointment of persons who were not qualified to be nominated. And as to the violation of Bye-law II.—that now under consideration—his argument was contemptuously brushed aside on the ground that the law itself was *ultra vires*. It went without saying that he and those who thought with him did not regard the pronouncement of Mr. Parker (an interested person in this matter) as authoritative, or as having the least weight whatever.

It was suggested at that committee meeting that it was open to him to apply to the Courts by a complicated and rarely-used process for a pronouncement on the validity of this bye-law, but it could hardly be expected that any single governor should put himself to the expense and trouble of justifying a law which was already on the books, which had been accepted without question for nearly half a century, which was a necessary bye-law, and one in force in all the hospitals whose rules he had been able to collect. At any rate he entertained no doubt himself as to the validity and usefulness of the bye-law, and having these views, he asked, at the committee meeting on April 21st, to whom (in his compulsory absence) he was indebted for the proposing of his own name as a member of the committee at the annual court, and finding that Mr. Bernard Parker was responsible, he desired to state in the plainest and most public manner possible that he declined to sit on the committee as the nominee of Mr. Parker or any of his fellow-raiders, or further to act on the committee with them as colleagues.

The issue was, he hoped, now made clear to the governors. Without notice, without any warning whatever, seven gentlemen with whom he had worked in harmony ever since his election to the committee had been ejected by votes manufactured for a particular purpose. He could not allow such tactics to pass without a public protest in the general interests not only of this but of all hospitals, or without endeavouring to save the Royal Orthopædic Hospital from a recurrence of such irregular proceedings by suggesting the strengthening of the laws, so that the regular subscribers might not be swamped by the votes of new-comers marshalled by a single force to gain a particular end.

Mr. H. H. MARKS, M.P., said he did not rise for the purpose of opposing the motion, because, so far as he was aware, there was no objection to increasing the time of qualification for a governor from six to twelve months, always assuming that there was a power on the part of the governors of the hospital to make such a rule. Upon that point he desired some guidance, as in the charter it was provided (in the second paragraph of page 3), without qualification, that each governor should be entitled to one vote at all elections. As to what had been said as to the election of the new committee at the annual court, he ventured to protest against the warmth which had been introduced into the discussion and to some of the terms which had been employed. Those who were acting with him had been referred to as interested parties. He hoped that all there were interested parties; they showed it by their presence there that day. He objected still more to the term "raiders," which had been applied to them. It was not, in his opinion, either in good temper or in good taste. It was perfectly true that at the last annual court, at which the now existing committee was elected, there were very exceptional circumstances. A deliberate and systematic attempt had been made to elect a sufficient number of governors to attend that meeting, in order that the management of the hospital might be taken out of the hands of those who had controlled it. No one was prepared to deny that; but he submitted that an act was to be judged by the motive that impelled it, as well as by the act itself. The circumstances had been briefly related in a circular issued by Mr. Bernard Parker, which did not in a single respect overstate the facts, and he challenged the old committee to controvert it in one single important particular. For years past the income of the hospital had been decreasing, the expenditure had shown an increase, and the hospital was in a most insanitary position. A grant from the Prince of Wales's Hospital Fund had been refused on this ground, and it had recently been found necessary to stop the reception of patients and to shut up a portion of the hospital on account of an outbreak of scarlet fever. If this had been due to accident; had they been taken by surprise; or if it had been owing to the prevalence of an epidemic, there would, of course, have been no condemnation necessary, but their case was that during the last three years no effective steps had been taken by the old committee to put the hospital in a satisfactory condition. When these facts were represented to him he had no hesitation in saying that if he could be of any service in rescuing the hospital he would very willingly do so. He had induced a number of friends to become governors, and eight of these had been elected on the committee in the hope that the introduction of new blood by this somewhat exceptional and even revolutionary method would have some result. These were the circumstances which had induced his friends to endeavour to bring a little life, activity, and expedition in carrying out the reforms in the institution which were admitted in the printed reports of the hospital to be needed. He submitted that it was not in a flourishing financial condition and in a very bad sanitary condition, and though he frankly admitted that the methods they had adopted were irregular, yet these steps were taken with a view to bring about a much-desired improvement. If they had erred in zeal it was due to a desire to assist where assistance was urgently required. He was a governor of not less than a dozen London hospitals, and he thought they would agree with him that the interests of the hospitals were the concern of every good citizen.

After considerable discussion with regard to various statements made and challenged by both sides, the motion was put to the meeting and adopted.

Mr. J. E. DROWER then proposed that Law XIV. should read, "That the committee of management shall consist of not less than twelve life, decennial, or annual governors, who at the date of their election to the committee shall have been governors of the hospital for a period of not less than twelve months, and who shall be elected annually at the annual court, with power to such court to add to their number, and three shall be a quorum." He warmly defended the action of the old committee, and traversed in detail many of the points referred to by Mr. Marks and in the printed circular of Mr. Bernard Parker. He explained the exceptional difficulties which the old committee had had in dealing with the situation, but said that they had arrived at a point when they had succeeded in obtaining an offer of £28,000 for the present site, and were proposing to submit to the governors a proposal for the sale of the present site and the transfer of the charity to some larger and more suitable premises in a neighbourhood less expensive, though equally convenient for the patients who take advantage of its benefits, when they were stopped by the election of the new governors. He characterised as gratuitously false the allegation in Mr. Parker's circular that from the date of the election of the new committee the old committee "had been endeavouring to frustrate the objects which the majority of the new committee had in view, namely, the better conduct of the business of the hospital, the increase of the revenue, and the question of the sale of the present site," and went on to say that they now realised that Mr. Reeves, one of their surgical officers, who had seemed to be working amicably and harmoniously with the old committee, was, with Mr. Marks and Mr. Parker, really the instigator of the present movement. It had been a bitter blow to him and his friends, just when they thought they saw a way out of all their difficulties, that their plans should have been upset as they were; he did not grudge his opponents the fruits of victory, but he was sure they would find them Dead Sea fruits—dust and ashes in the mouth.

Mr. TITUS BARHAM seconded the motion.

Mr. BERNARD PARKER then entered into a defence of his circular which Mr. DROWER had attacked, and a long and at times acrimonious discussion ensued, but eventually the motion was agreed to, as was also a resolution that the following new law be added, and numbered Law XIVA.: "That no governor shall be proposed for election on the committee, unless the governor desirous of nominating him shall give notice thereof, in writing, to the secretary, at least fourteen days prior to the day of election; but this notice shall not be requisite in the case of a governor seeking or being proposed for re-election."

Mr. Alderman BELL then proposed that the following new law be added and numbered Law XIVB.: "That no acting medical officer of the hospital shall be eligible for election as a member of the committee of management." He said that if any justification for this proposal were needed it had been supplied by what had taken place that day. For those who could read between the lines it was obvious that behind Mr. Marks and Mr. Parker there was the moving spirit of the whole party—Mr. H. A. Reeves. Mr. Reeves was supposed to be a member of the medical staff working in concert with the committee. Mr. Reeves had strong views against the unanimous decision of the old committee that it was necessary for the hospital to be removed from its present site, and in order to defeat that object he had promoted the present opposition. In his (Mr. Alderman Bell's) judgment the best thing that could happen to the institution would be to start afresh on a new site and with a wholly new medical staff. It was generally recognised that it was very undesirable for members of the medical staff to be on a committee of management. It was so at the Hospital for Diseases of the Throat in Golden Square, and it ought to be so at the Orthopædic Hospital.

Mr. Marks said that in view of the personal attack upon Mr. Reeves made by Mr. Alderman Bell, he should not be justified in preserving the attitude of benevolent neutrality he had maintained with regard to the other bye-laws proposed. Mr. Bell had made a direct personal attack on a professional gentleman who was held in high esteem in his profession. In his opinion it was essential for a representative of the medical staff to be on the committee under the circumstances of the hospital. He should therefore oppose the motion, for he would be no party to the passing of a resolution directed against those to whom the hospital owed a debt of gratitude.

On the motion being put to the meeting the voting was 12 for and 25 against; but on the proxies held by both sides being counted the Chairman gave the numbers as 60 for and 45 against, with 5 votes cast against disqualified by irregularity as to signature. The motion was therefore declared carried.

In proposing a vote of thanks to the President for taking the chair, Sir Walter Gilbey said that he and his colleagues felt they could not go on with the business of the hospital when it had been, as a newspaper phrased it, "captured" by Mr. Marks and his asscciates. They were sorry to sever their connection with the hospital, but they felt they had no alternative whilst it was under its present management. He begged, therefore, to hand to Lord Wantage a letter addressed to him as president by his four colleagues, Mr. Alderman Bell, Sir Ernest Clarke, Mr. Drower, Mr. Samuel Studd, and himself, tendering their resignations as members of the committee, to be dealt with as his Lordship should think fit.

Mr. Alderman Bell having seconded the vote of thanks,

Lord Wantage said that he felt it his duty to accept the resignations tendered to him. For himself, he recognised that his continuing in the office of president would under all the circumstances, be of very little avail, and therefore he must with great goodwill to the hospital, tender his own resignation as president.

The meeting then separated.

